# Supplementary material for: Untranslated Regions of a Segmented Kindia Tick Virus Genome Are Highly Conserved and Contain Multiple Regulatory Elements for Viral Replication
Source: Microorganisms. 2024 Jan 23;12(2):239. doi: 10.3390/microorganisms12020239 (PMC10893285; doi:10.3390/microorganisms12020239)
Supplement: Supplementary file 1 [file microorganisms-12-00239-s001.zip › microorganisms-2832257-supplementary.pdf]

Supplementary Material

Table S1. Oligonucleotides used for KITV PCR.

Primer	Sequence	T°C
Segment 1		
S1-1F	GCCCTTGACTGCAAGTGCAAA	57,6
S1-1R	TATCTACGTGCCAGGGCTTGAC	55,7
S1-2F	TGAAGAACGTCAAGCCCTGG	59,8
S1-2R	GCTGACCCACGAACCTGTTT	60,5
S1-3F	AAAAGAAGGGCTCTGAGGGC	59,5
S1-3R	CTTATACAGGCCCTGTCCCG	59,2
S1-4F	GAAGTGCGGATGGAGCGTAG	60,5
S1-4R	ACCTGTGGGAGCAGAAGGAT	60,1
S1-5F	CATAGTGGACGTGAAAGAAATATCC	57,4
S1-5R	TTTTACTTTTTCCCCCAATTCAGTTT	57,9
Segment 2		
S2-1F	GCCGGTGGCAAGTGGCATACATC	61,2
S2-1R	GACCACTGCCTGGACCTTCTCTGC	62,0
S2-2F	GAACAAAAAAAATAGAGAAAGAAACTGCTA	57,9
S2-2R	GGAAGGTTGTGTTCATGTGC	57,6
S2-3F	CGAGGACGCGAAATATGCC	58,7
S2-3R	CTCTGATGGTGAGCCTATGC	57,4
S2-4F	AACTTTGGGAGTGACCAGGG	59,1
S2-4R	GATAAGGCCGTCAGAGCGAG	59,9
S2-5F	CAGGGACGAGACATTGCCAA	59,9
S2-5R	CCGTGGAGTAGTGGACCGTA	60,3
S2-6F	ACCCACCATTCTCACTCGCA	61,0
S2-6R	GGGGCTTTAGCACTTGCCA	60,2
S2-7F	TCTATCTTATGCTATGACGCCCG	55,7
S2-7R	CCCTTCCGGATCCTCCTAGCTCA	60,1
Segment 3		
S3-1F	GCTGAGACTGCAAGTGCACA	60,7
S3-1R	CGGTCCGATGTGCTCAAAGA	60,0
S3-2F	AATTGGAGAGGCAGAGGGGA	59,8

S3-2R	GACCTTGTTGGACCAGGTCA	59,1
S3-3F	GGCAACTCATGACCTGGTCC	60,3
S3-3R	AGGACCACTGTGGCGTAGT	60,1
S3-4F	GCTAAACAGATCAGGCAGCTCA	60,2
S3-4R	TGCTCACGGCTTCTTCTTCC	59,9
S3-5F	GCGATGTATAACGTTGAAGGCG	59,9
S3-5R	TCGGTCGCTTCAACTACAGACT	61,0
Segment 4		
S4-1F	CTCTGAGTTGCAAGTGCTATAGC	59,0
S4-1R	TTTCCGGGTATAGTCTTGGTAGC	59,2
S4-2F	CGTGTATGCGGCTACCAAGA	59,8
S4-2R	GAATCCAGTGGTACGTCCCG	59,8
S4-3F	CCCTACCAGGCCTGATACGA	60,0
S4-3R	TAGTAGCGGGCCAGGTTGTA	59,9
S4-4F	GCGGAGAGAGAGAAAACGCA	60,0
S4-4R	ACAGGTTCACGAACACAGCC	60,4
S4-5F	GGCAACAATAGTGGGAATCGC	59,5
S4-5R	TCCGGAGTATGGAACGAGATCA	60,0

Table S2. Amino acid substitutions between isolates KITV/2018/1, KITV/2018/2 and KITV/2017/1. Amino acid substitutions relative to KITV/2018/1 and KITV/2018/2 are highlighted in blue.

Amino acid position	Region	Isolate KITV/2018/1	Isolate KITV/2018/2	Isolate KITV/2017/1
135	Segment 1 (NS5)	Val	Ile	Val
217		Ala	Val	Ala
233		Lys	Arg	Lys
277		Arg	Arg	Ser
610		His	His	Arg
674		Ala	Thr	Ala
747		Cys	Cys	Tyr
908		Gln	Arg	Gln
76	Segment 2 (VP1)	Phe	Phe	Ser
89		His	His	Pro
103		Gln	Gln	His
160		Phe	Phe	Leu
247		Val	Val	Gly
368		Ile	Val	Ile

380		Thr	Thr	Ala
435		Glu	Glu	Lys
465		Gly	Gly	Ser
604		Val	Val	Ala
622		Ala	Thr	Ala
236	Segment 3 (NS3)	Val	Val	Ala
247		Val	Val	Ala
291		Ile	Val	Val
379		Val	Val	Ala
459		Lys	Lys	Asn
474		Arg	Arg	Ser
476		Ser	Phe	Ser
604		Gly	Gly	Arg
625		Lys	Arg	Lys
673		Glu	Gln	Glu
146	Segment 4 (VP2)	Ser	Ser	Asn
6	Segment 4 (VP3)	Asp	Asp	Val
18		Met	Met	Ile
79		Asn	Ser	Asn
208		Lys	Arg	Lys
338		Val	Val	Gly
353		Phe	Phe	Leu

Table S3. Location of putative UAG binding sites for MSI1 protein in the 3′ UTRs of KITV and MGTV. Red color – conserved site between Y-3 and Y-2 structures; yellow color – conserved site in the stem of the Y-2 structure; green color - conserved site in the stem of the Y-1 structure.

The name of the KITV and MGTV isolate, the place of isolation	Coordinates of the location of sites			
	Segment 1	Segment 2	Segment 3*	Segment 4
KITV/2017/1, Guinea, Africa	43, 48, 80	202, 373	12, 43, 94, 104, 138	51, 123
KITV/2018/1, Guinea, Africa		167, 268, 338	12, 43, 94, 104, 138	
KITV/2018/2, Guinea, Africa		167, 268, 338	43, 94, 104, 138	
MGTV Yunnan2016, China, Asia		168, 261	94, 104, 138	

Note: *- results for this region are not discussed in this work, since the region is undersequenced.

Table S4. Nucleotide substitutions in the 5’ and 3’ UTRs between different KITV isolates.

	Nucleotide position	MK673133.1 KITV/2017/1	MW341206.1 KITV2018/1	MW341210.1 KITV/2018/2	OP612399.1	OP612400.1	OP612401.1	OP612402.1	OP612403.1	OP612404.1	OP612405.1	OP612406.1	OP612407.1	OP612408.1	OP612409.1	OP612410.1	OP612411.1	OP612412.1	OP612413.1
Segment 1		5’UTR																	
	1-13	GTTAAAA GAGGCC	------------	------------	------------	------------	------------	------------	------------	------------	------------	------------	------------	------------	------------	------------	------------	------------	------------
		3’UTR																	
	4	C	C	C	T	T	T	T	T	T	T	T	T	T	T	T	T	C	T
	120-127	--------	AAGTA AAA	AAGTA AAA	AAGTA	AAGTA	AAGTA	AAGTA	AAGTA	AAGTA	AAGTA	AAGTA	AAGTA	AAGTA	AAGTA	AAGTA	AAGTA	AAGTA	AAGTA
Segment 2		MK673134.1 KITV/2017/1	MW341207.1 KITV2018/1	MW341211.1 KITV/2018/2	OP612414.1	OP612415.1	OP612416.1	OP612417.1	OP612418.1	OP612419.1	OP612420.1	OP612421.1	OP612422.1	OP612423.1	OP612424.1	OP612425.1	OP612426.1	OP612427.1	OP612428.1
		5’UTR																	
	1-32	GCCGGTGGCAAGTGCA TACATCGACAACGAAT	GCCGGTGGCAAGTGCA TACATCGACAACGAAT	GCCGGTGGCAAGTGCA TACATCGACAACGAAT	------------------------	------------------------	------------------------	------------------------	------------------------	------------------------	------------------------	------------------------	------------------------	------------------------	------------------------	------------------------	------------------------	------------------------	------------------------
	93	T	-	-	-	-	-	-	-	-	-	-	-	-	-	-	-	-	-
	97	A	-	-	-	-	-	-	-	-	-	-	-	-	-	-	-	-	-
	104	A	A	T	T	T	T	T	T	T	T	T	T	T	T	T	T	T	T
		3’UTR																	
	44	C	T	C	C	C	C	C	C	C	C	C	C	C	C	C	C	C	C
	54	T	T	C	T	T	T	T	T	T	T	T	T	T	T	T	T	T	T
	57	A	A	A	G	G	G	G	G	G	G	G	G	G	G	G	G	G	G
	70	C	C	C	C	C	C	C	C	C	C	A	A	A	A	C	C	C	C
	74	G	T	T	T	T	T	T	T	T	T	T	T	T	T	T	T	T	T

			Segment 3																		
			5'UTR														3'UTR				
	301	75-109	14	63	66	67-76	77	79	84	85	86	91	106	108	109	110	2	3	11	13	21
MK673135.1 KITV/2017/1	G	GAAAGGCATCGGACACTC GAACACGGAACGCACCA	T	C	A	----------	-	G	A	-	-	A	C	T	T	G	C	C	G	A	T
MW341208.1 KITV/2018/1	A	-----------------------------------	C	T	G	AAAGAAA GGA	A	A	C	T	T	G	T	C	A	A	C	C	G	T	T
MW341212.1 KITV/2018/2	A	-----------------------------------	C	T	A	----------	A	A	C	T	T	A	T	T	A	A	T	T	C	T	C
OP612443.1	A	-----------------------------------	C	T	G	----------	-	A	C	T	T	A	T	C	A	A	C	C	G	T	C
OP612442.1	A	-----------------------------------	C	T	G	----------	-	A	C	T	T	A	T	C	A	A	C	C	G	T	C
OP612441.1	A	-----------------------------------	C	T	G	----------	-	A	C	T	T	A	T	C	A	A	C	C	G	T	C
OP612440.1	A	-----------------------------------	C	T	G	----------	-	A	C	T	T	A	T	C	A	A	C	C	G	T	C
OP612439.1	A	-----------------------------------	C	T	A	----------	-	A	A	T	T	A	T	A	A	A	C	C	G	T	C
OP612438.1	A	-----------------------------------	C	T	G	----------	-	A	C	T	T	A	T	C	A	A	C	C	G	T	C
OP612437.1	A	-----------------------------------	C	T	G	----------	-	A	C	T	T	A	T	C	A	A	C	C	G	T	C
OP612436.1	A	-----------------------------------	C	T	G	----------	-	A	C	T	T	A	T	C	A	A	C	C	G	T	C
OP612435.1	A	-----------------------------------	C	T	A	----------	-	A	A	T	T	A	T	A	A	A	C	C	G	T	C
OP612434.1	A	-----------------------------------	C	T	A	----------	-	A	A	T	T	A	T	A	A	A	C	C	G	T	C
OP612433.1	A	-----------------------------------	C	T	A	----------	-	A	A	T	T	A	T	A	A	A	C	C	G	T	C
OP612432.1	A	-----------------------------------	C	T	G	----------	-	A	C	T	T	A	T	C	A	A	C	C	G	T	C
OP612431.1	A	-----------------------------------	C	T	G	----------	-	A	C	T	T	A	T	C	A	A	C	C	G	T	C
OP612430.1	A	-----------------------------------	C	T	G	----------	-	A	C	T	T	A	T	C	A	A	C	C	G	T	C
OP612429.1	A	-----------------------------------	C	T	G	----------	-	A	C	T	T	A	T	C	A	A	C	C	G	T	C

Segment	Strain	61	62	144	145-178	5'UTR 35	5'UTR 89	3'UTR 14	3'UTR 245-247
Segment 4	MK673136.1 KITV/2017/1	C	G	-	-------------------------	T	C	C	---
	MW341209.11 KITV/2018/1	A	T	-	TGGAACATAAAAGTCTGTAGTTGAAGCGACC GAA	T	C	C	GGA
	MW341213.1 KITV/2018/2	A	T	-	-------------------------	T	T	C	GGA
	OP612458.1	A	T	A	TGGAACATAAAAGTCTGTAGTTGAAGCGACC	C	C	T	GGA
	OP612457.1	A	T	A	TGGAACATAAAAGTCTGTAGTTGAAGCGACC	T	C	T	GGA
	OP612456.1	A	T	A	TGGAACATAAAAGTCTGTAGTTGAAGCGACC	T	C	T	GGA
	OP612455.1	A	T	A	TGGAACATAAAAGTCTGTAGTTGAAGCGACC	C	C	T	GGA
	OP612454.1	A	T	A	TGGAACATAAAAGTCTGTAGTTGAAGCGACC	C	C	T	GGA
	OP612453.1	A	T	A	TGGAACATAAAAGTCTGTAGTTGAAGCGACC	T	C	T	GGA
	OP612452.1	A	T	A	TGGAACATAAAAGTCTGTAGTTGAAGCGACC	T	C	T	GGA
	OP612451.1	A	T	A	TGGAACATAAAAGTCTGTAGTTGAAGCGACC	T	C	T	GGA
	OP612450.1	A	T	A	TGGAACATAAAAGTCTGTAGTTGAAGCGACC	T	C	T	GGA
	OP612449.1	A	T	A	TGGAACATAAAAGTCTGTAGTTGAAGCGACC	T	C	T	GGA
	OP612448.1	A	T	A	TGGAACATAAAAGTCTGTAGTTGAAGCGACC	T	C	T	GGA
	OP612447.1	A	T	A	TGGAACATAAAAGTCTGTAGTTGAAGCGACC	C	C	T	GGA
	OP612446.1	A	T	A	TGGAACATAAAAGTCTGTAGTTGAAGCGACC	C	C	T	GGA
	OP612445.1	A	T	A	TGGAACATAAAAGTCTGTAGTTGAAGCGACC	C	C	T	GGA
	OP612444.1	A	T	A	TGGAACATAAAAGTCTGTAGTTGAAGCGACC	C	C	T	GGA

Table S5. Conserved regions in 5′ UTR of the KITV Segment 1.

Name of the virus (number of strains)	Motifs 1, 2, 4	Motif 1	Motif 2	Motif 4	Motifs 1, 2	Motifs 1, 4	Motifs 2, 4	Motifs characteristic of KITV were not found
KITV (18)	18 (100%)	0	0	0	0	0	0	0
JMTV (54)	32 (59,26%)	2 (3,70%)	0	0	5 (9,26%)	13 (24,07%)	0	2 (3,70%)
MGTV (3)	3 (100%)	0	0	0	0	0	0	0

Alongshan virus (8)	0	0	5 (62,5%)	0	0	0	0	3 (37,5%)
Yanggou virus (6)	0	0	0	2 (33,33%)	0	0	4 (66,67%)	0
Takachi virus (5)	0	0	0	0	0	0	0	5 (100%)
Harz mountain virus (7)	0	0	7 (100%)	0	0	0	0	0

Table S6. Conserved regions in 3′ UTR of the KITV Segment 1.

Name of the virus (number of strains)	Motifs 1, 3	Motif 1	Motifs characteristic of KITV were not found
KITV (18)	18 (100%)	0	0
JMTV (79)	74 (93,67%)	4 (5,1%)	1 (1,27%)
MGTV (3)	3 (100%)	0	0
Alongshan virus (10)	0	10 (100%)	0
Yanggou virus (6)	0	6 (100%)	0
Takachi virus (5)	0	5 (100%)	0
Harz mountain virus (7)	0	7 (100%)	0

Table S7. Conserved regions in 5′ UTR of the KITV Segment 2.

Name of the virus (number of strains)	Motifs 1, 2, 3, 4	Motifs 1, 2, 3	Motifs 1, 2	Motif 4	Motifs 2, 4	Motifs 1, 2, 4	Motifs characteristic of KITV were not found
KITV (18)	1 (5,56%)	2 (11,11%)	15 (83,33%)	0	0	0	0
JMTV (81)	35 (43,21%)	1 (1,23%)	0	0	30 (37,04%)	14 (17,28%)	1 (1,23%)

MGTV (3)	1 (33,33%)	0	0	0	0	0	2 (66,67%)
Alongshan virus (7)	0	0	0	1 (14,29%)	0	0	6 (85,71%)
Yanggou virus (3)	0	0	0	0	0	0	3 (100%)
Sichuan tick virus (3)	0	2 (66,67%)	1 (33,33%)	0	0	0	0
Harz mountain virus (5)	0	0	0	5 (100%)	0	0	0
SCWL (1)	0	1 (100%)	0	0	0	0	0

Table S8. Conserved regions in 3′ UTR of the KITV Segment 2.

Name of the virus (number of strains)	Motifs 1, 2, 3, 4, 5	Motifs 1, 2, 3, 5	Motifs 1, 2, 3	Motif 1	Motifs 1, 4	Motif 4	Motifs 2, 3	Motifs 1, 3	Motif 2	Motifs characteristic of KITV were not found
KITV (18)	18 (100%)	0	0	0	0	0	0	0	0	0
JMTV (82)	44 (53,66%)	2 (2,44%)	14 (17,07 %)	0	0	0	8 (9,76%)	1 (1,22%)	4 (4,88%)	9 (10,98%)
MGTV (3)	1 (33,33%)	2 (66,67%)	0	0	0	0	0	0	0	0
Alongshan virus (9)	0	0	0	5 (55,56%)	1 (11,11%)	0	0	0	0	3 (33,33%)
Yanggou virus (3)	0	0	0	0	0	1 (33,33%)	0	0	0	2 (66,67%)
Sichuan tick virus (3)	1 (33,33%)	1 (33,33%)	1 (33,33%)	0	0	0	0	0	0	0
Harz mountain virus (5)	0	0	0	0	5 (100%)	0	0	0	0	0
SCWL (1)	0	0	1 (100%)	0	0	0	0	0	0	0

Table S9. Conserved regions in 5′ UTR of the KITV Segment 3.

Name of the virus (number of strains)	Motifs 1, 2	Motif 1	Motif 2	Motifs characteristic of KITV were not found
KITV (18)	18 (100%)	0	0	0
JMTV (74)	42 (56,76%)	2 (2,70%)	18 (24,32%)	12 (16,22%)
MGTV (3)	3 (100%)	0	0	0
Alongshan virus (8)	0	0	0	8 (100%)
Yanggou virus (3)	0	0	0	3 (100%)
Sichuan tick virus (3)	3 (100%)	0	0	0
Harz mountain virus (7)	0	0	0	7 (100%)
SCWL (1)	1 (100%)	0	0	0

Table S10. Conserved regions in 5′ UTR of the KITV Segment 4.

Name of the virus (number of strains)	Motifs 1, 2, 3	Motifs 2, 3	Motifs 1, 2	Motif 1	Motif 2	Motif 3	Motifs characteristic of KITV were not found
KITV (18)	18 (100%)	0	0	0	0	0	0
JMTV (71)	42 (59,15%)	1 (1,41%)	14 (19,72%)	2 (2,82%)	5 (7,04%)	3 (4,23%)	4 (5,63%)
MGTV (3)	3 (100%)	0	0	0	0	0	0
Alongshan virus (8)	0	0	0	0	0	8 (100%)	0
Yanggou virus (3)	0	0	0	0	0	1 (33,33%)	2 (66,67%)
Sichuan tick virus (3)	3 (100%)	0	0	0	0	0	0
Harz mountain virus (7)	0	0	0	0	0	7 (100%)	0
SCWL (1)	1 (100%)	0	0	0	0	0	0

Table S11. Conserved regions in 3′ UTR of the KITV Segment 4.

Name of the virus (number of strains)	Motifs 1, 2, 3, 4, 5, 6	Motifs 1, 2, 3, 5, 6	Motifs 1, 2, 5, 6	Motifs 2, 5, 6	Motifs 1, 4	Motif 5	Motifs 1, 4, 6	Motif 1	Motifs characteristic of KITV were not found
KITV (18)	18 (100%)	0	0	0	0	0	0	0	0
JMTV (93)	51 (54,84%)	10 (10,75%)	24 (25,81%)	3 (3,23%)	1 (1,08%)	3 (3,23%)	0	0	1 (1,08%)
MGTV (3)	3 (100%)	0	0	0	0	0	0	0	0
Alongshan virus (8)	0	0	0	0	1 (12,5%)	0	7 (87,5%)	0	0
Yanggou virus (3)	0	0	0	0	0	0	0	3 (100%)	0
Sichuan tick virus (3)	3 (100%)	0	0	0	0	0	0	0	0
Harz mountain virus (7)	0	0	0	0	7 (100%)	0	0	0	0
SCWL (1)	1 (100%)	0	0	0	0	0	0	0	0

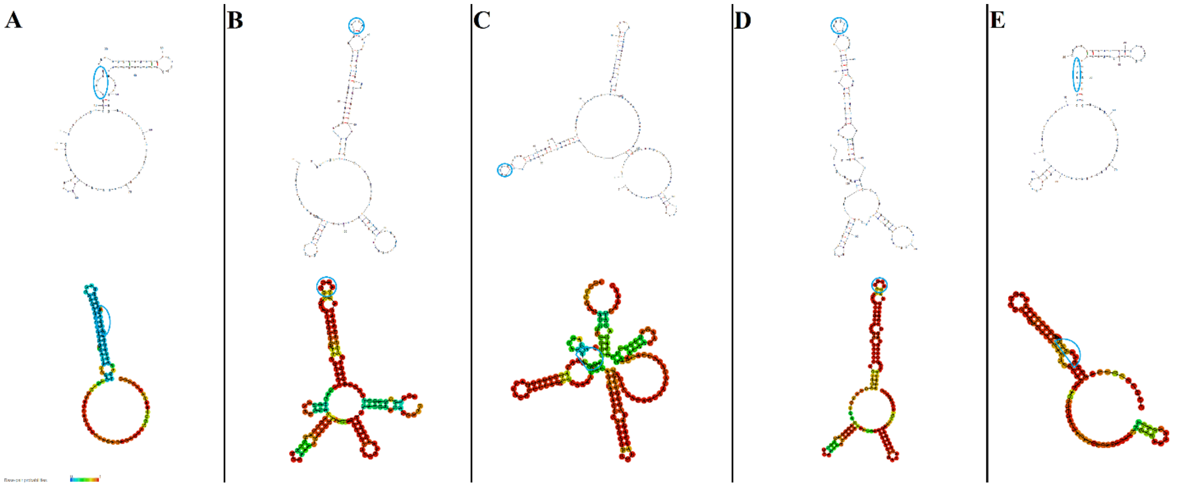

Figure S1. Models of secondary structures of the 5’ UTR of the first segment of viruses (A) MGTV, (B) ALSV, (C) YGTV, (D) HMV, (E) JMTV. The area of the highly conservative sequence 5′-CAAGUG-3′ is marked in blue. The models from the top were obtained in the UNA MFOLD 3.6 program, from the bottom in the Vienna RNA Fold program.

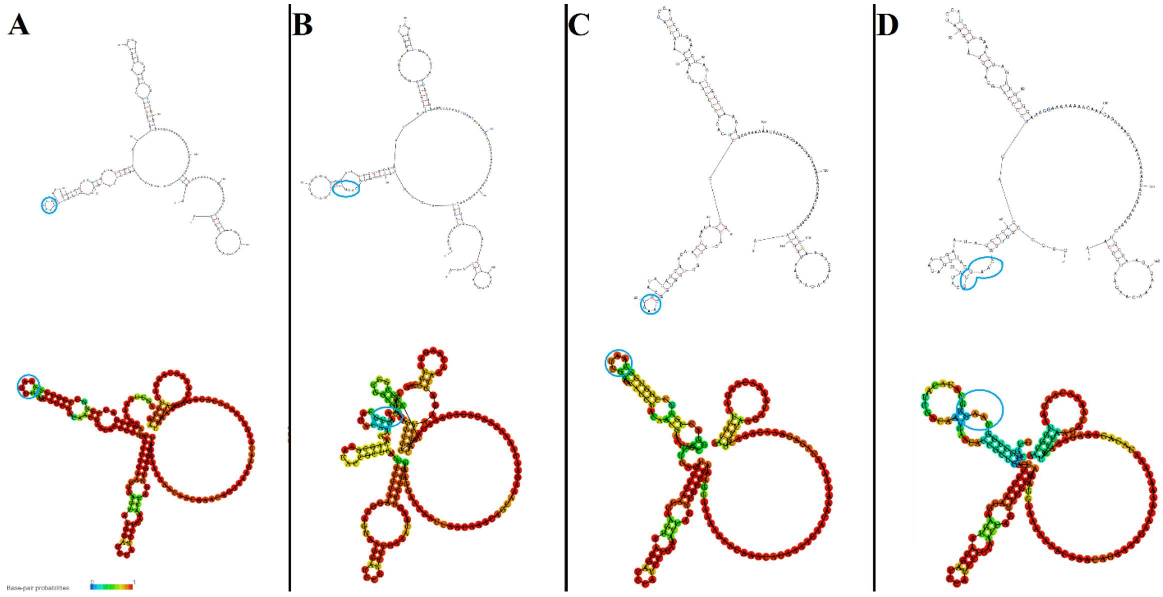

Figure S2. Models of secondary structures of the 5’ UTR of the second segment of viruses (A) MGTV, (B) JMTV, (C) SCWLTV, (D) STV. The area of the highly conservative sequence 5′-CAAGUG-3′ is marked in blue. The models from the top were obtained in the UNA MFOLD 3.6 program, from the bottom in the Vienna RNA Fold program.

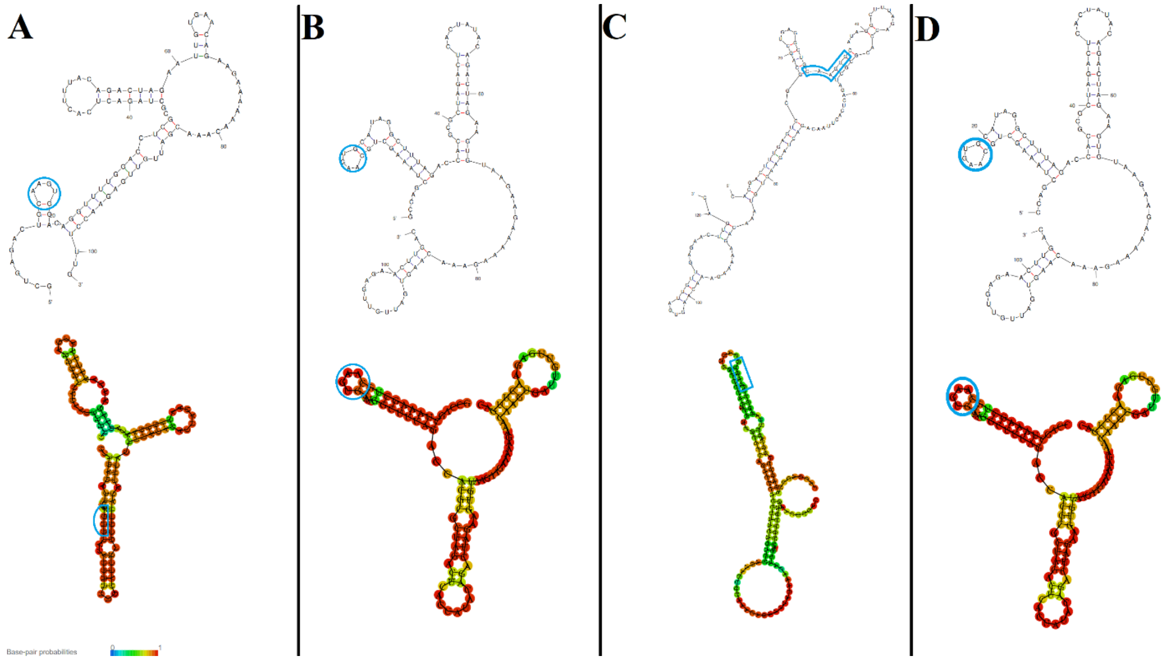

Figure S3. Models of secondary structures of the 5’ UTR of the third segment of viruses (A) MGTV, (B) STV, (C) JMTV, (D) SCWLT. The area of the highly conservative sequence 5′-CAAGUG-3′ is marked in blue. The models from the top were obtained in the UNA MFOLD 3.6 program, from the bottom in the Vienna RNA Fold program.

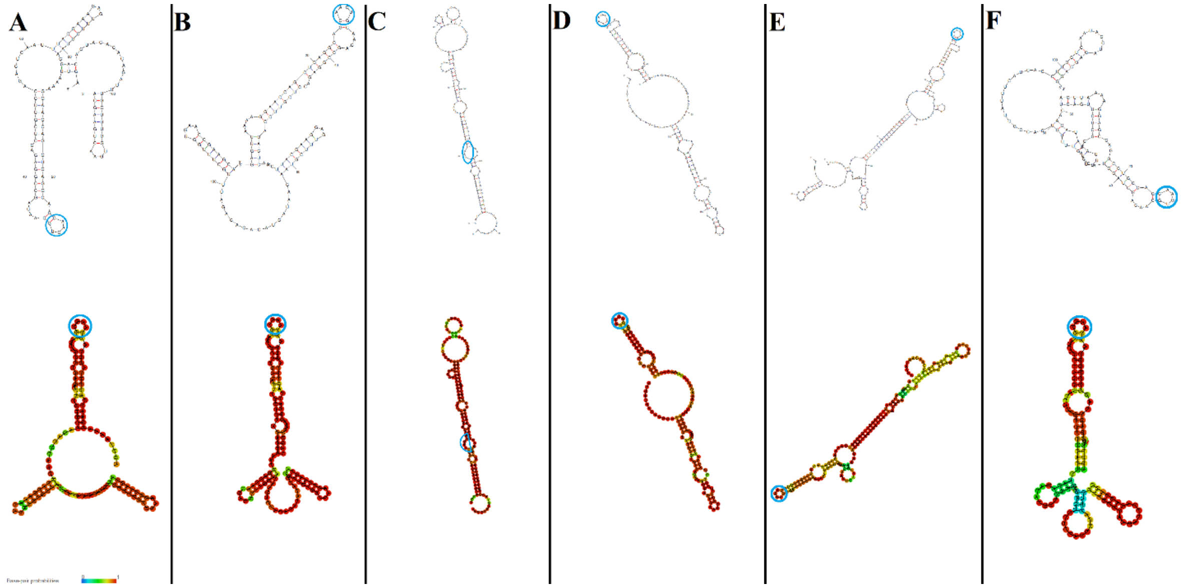

Figure S4. Models of secondary structures of the 5' UTR of the fourth segment of viruses (A) ALSV, (B) HMV, (C) SCWLTV, (D) STV, (E) JMTV, (F) YGTV. The area of the highly conservative sequence 5′-CAAGUG-3′ is marked in blue. The models from the top were obtained in the UNA MFOLD 3.6 program, from the bottom in the Vienna RNA Fold program.

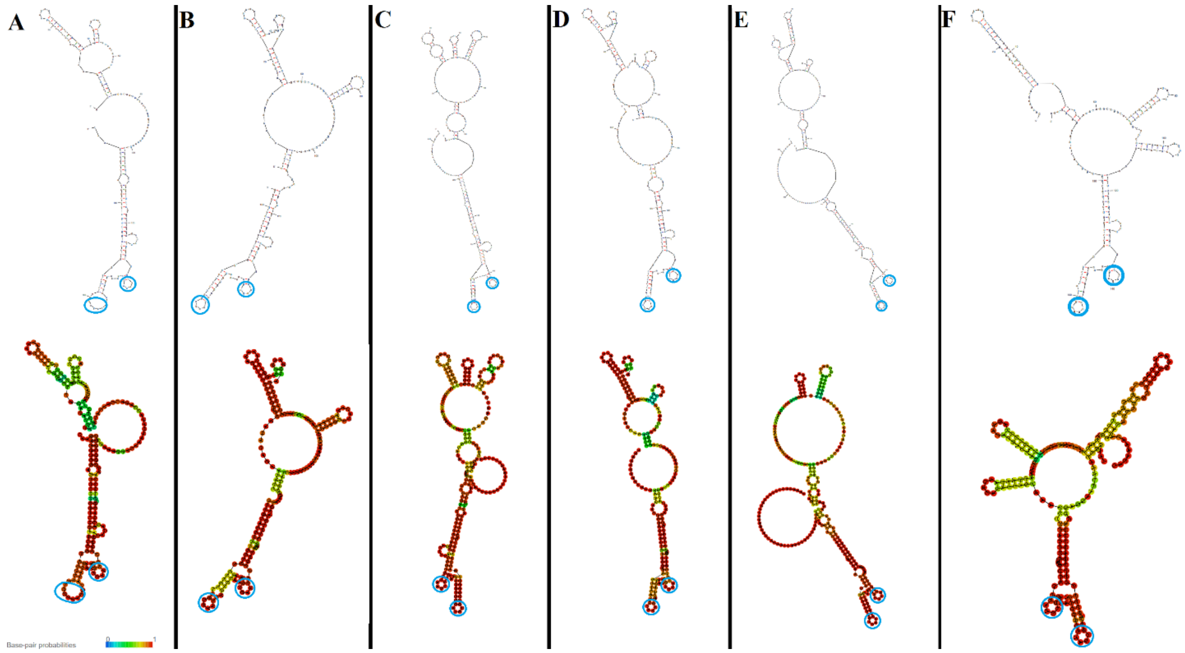

Figure S5. Models of secondary structures of the 3' UTR of the first segment of viruses (A) TAKV, (B) ALSV, (C) MGTV, (D) HMV, (E) JMTV, (F) YGTV. The area of the highly

conservative sequence 5′-CAAGUG-3′ is marked in blue. The models from the top were obtained in the UNA MFOLD 3.6 program, from the bottom in the Vienna RNA Fold program.

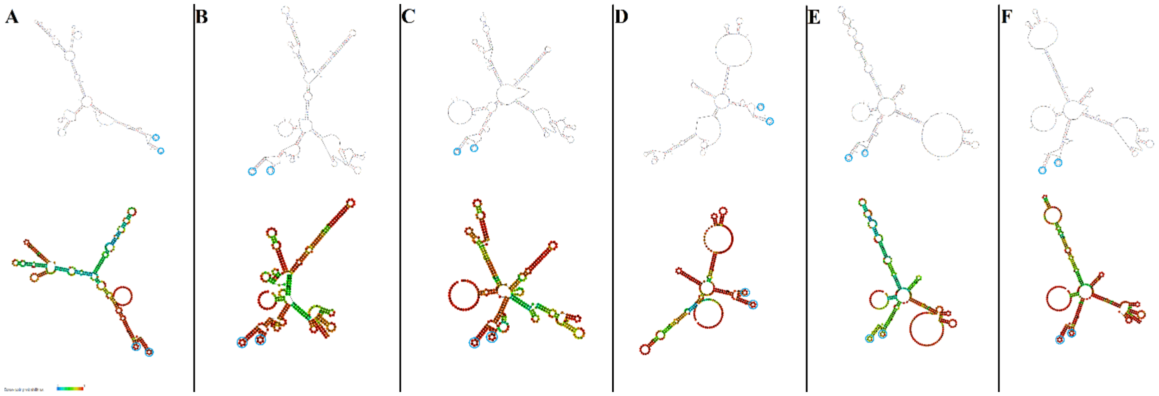

Figure S6. Models of secondary structures of the 3’ UTR of the second segment of viruses (A) MGTV, (B) HMV, (C) ALSV, (D) YGTV, (E) STV, (F) JMTV. The area of the highly conservative sequence 5′-CAAGUG-3′ is marked in blue. The models from the top were obtained in the UNA MFOLD 3.6 program, from the bottom in the Vienna RNA Fold program.

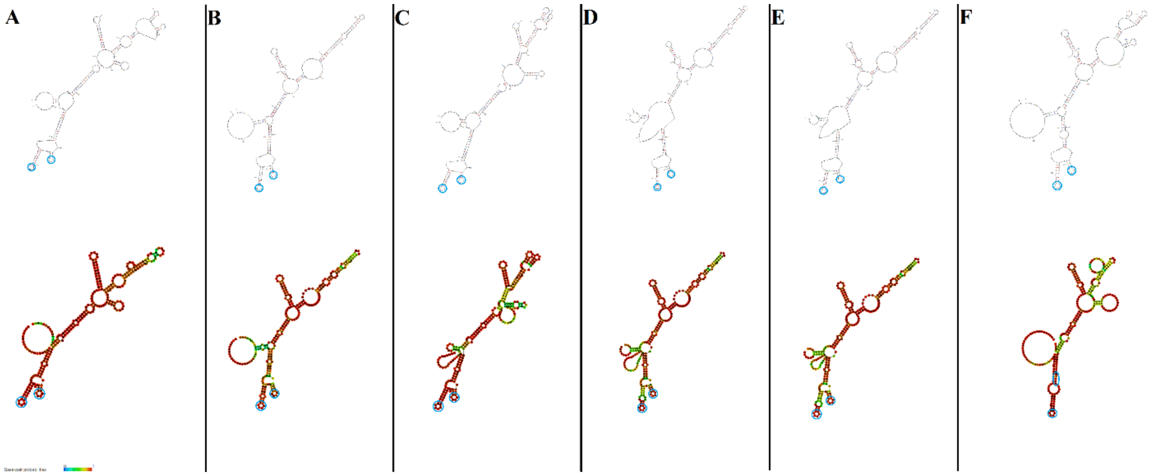

Figure S7. Models of secondary structures of the 3’ UTR of the fourth segment of viruses (A) ALSV, (B) MGTV, (C) HMV, (D) SCWLTV, (E) STV, (F) JMTV. The area of the highly conservative sequence 5′-CAAGUG-3′ is marked in blue. The models from the top were obtained in the UNA MFOLD 3.6 program, from the bottom in the Vienna RNA Fold program.

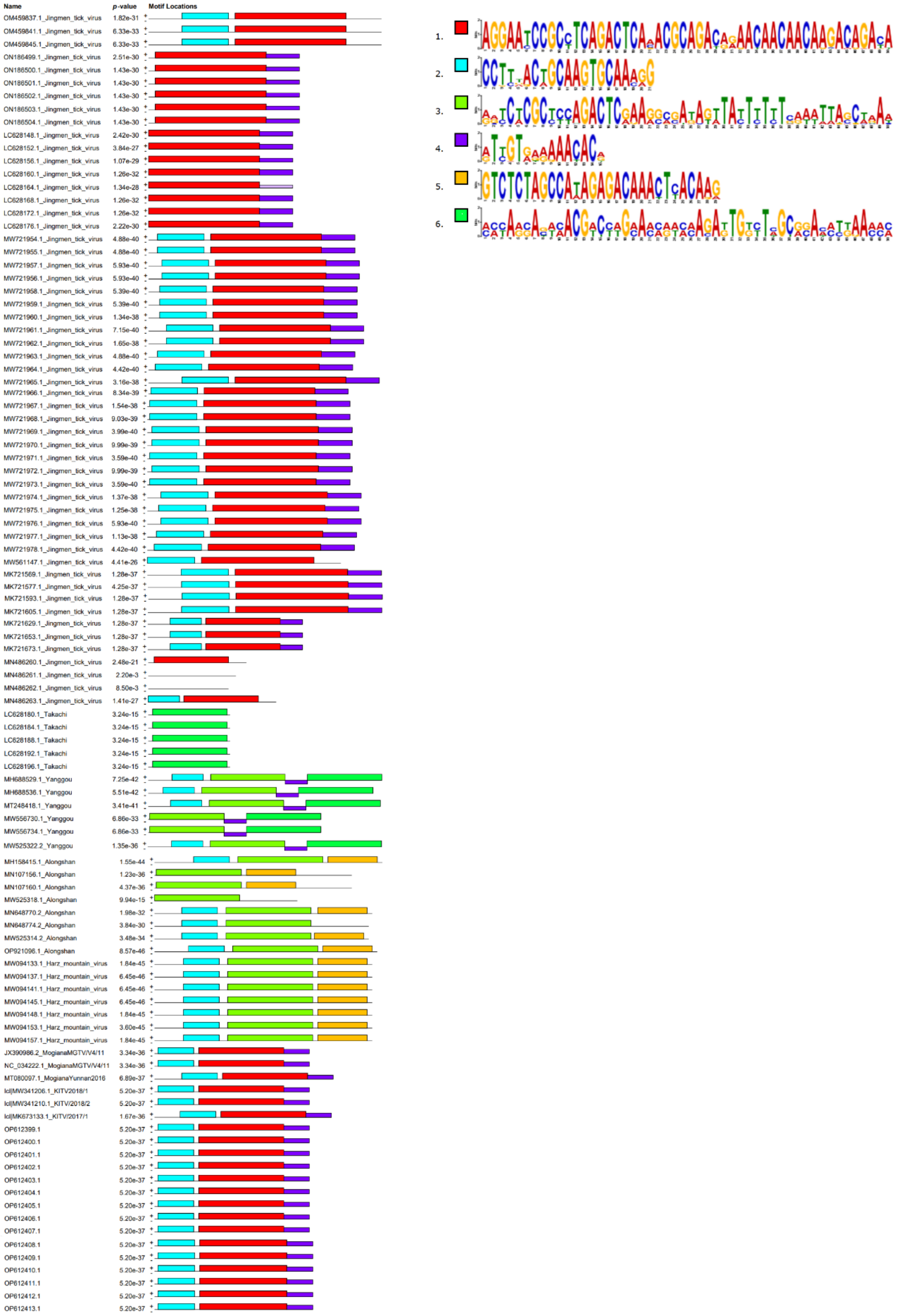

Figure S8. Conservative motifs in the 5′ UTR of segment 1 of segmented flavi-like viruses.

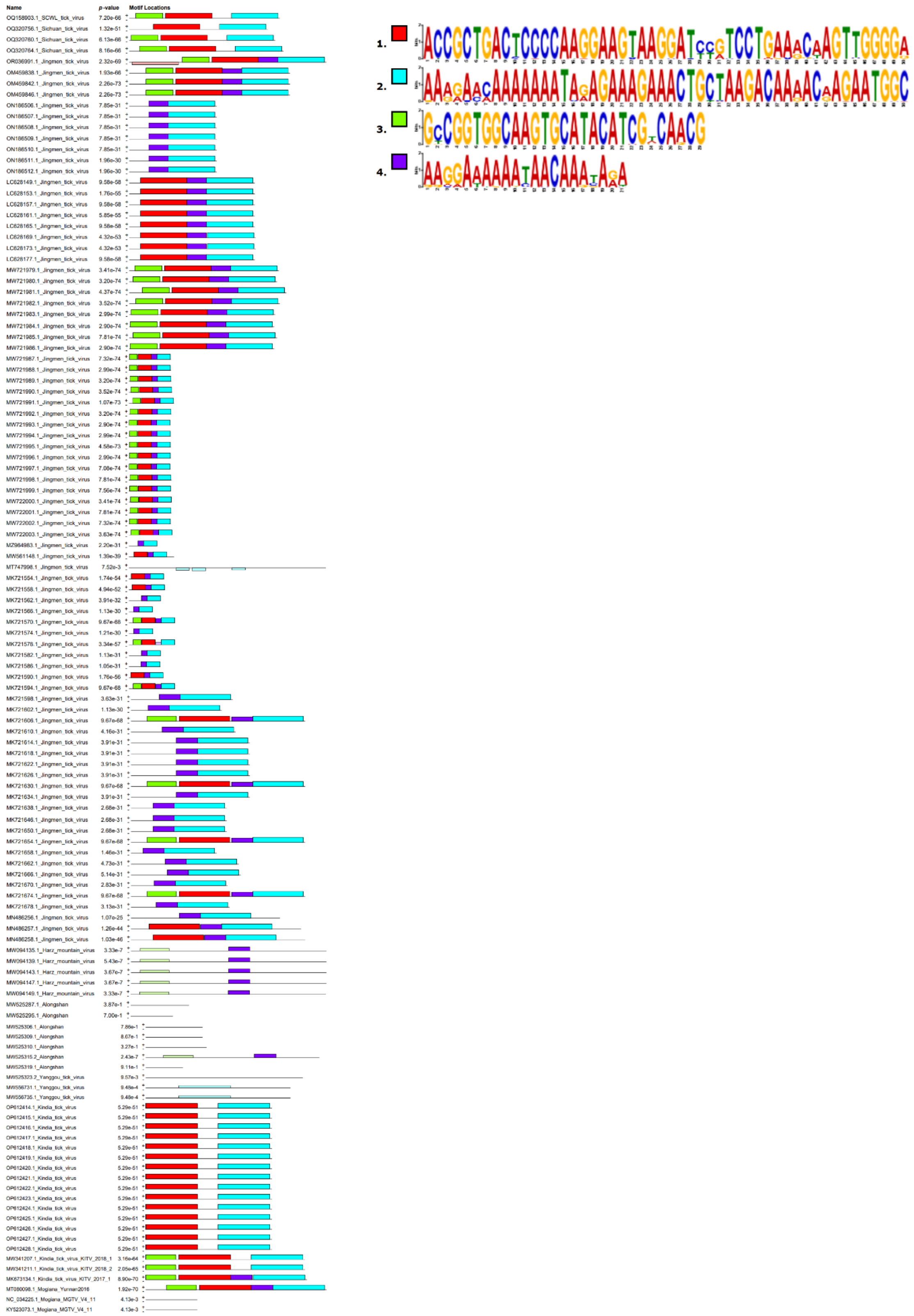

Figure S9. Conservative motifs in the 5′ UTR of segment 2 of segmented flavi-like viruses.

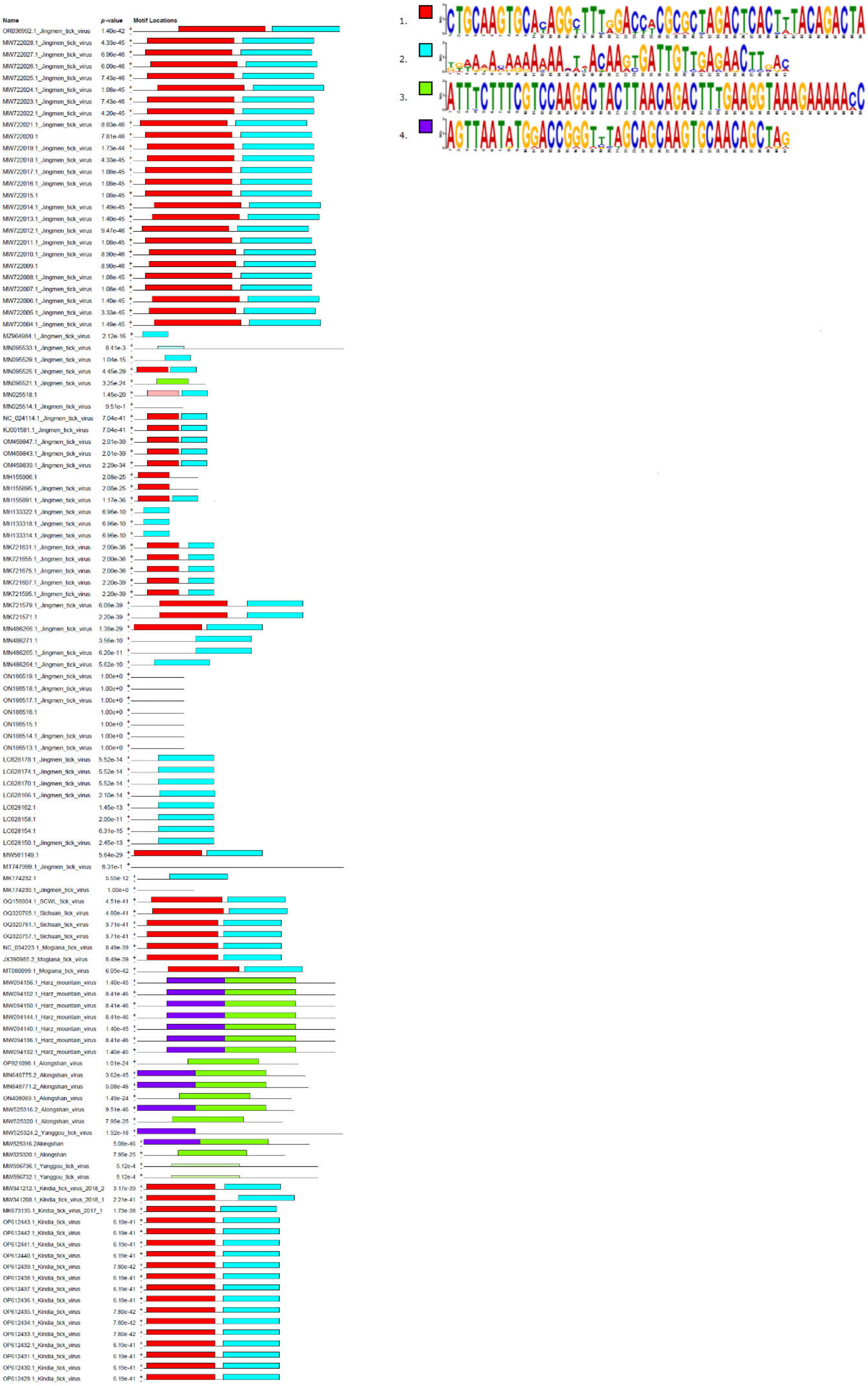

Figure S10. Conservative motifs in the 5′ UTR of segment 3 of segmented flavi-like viruses.

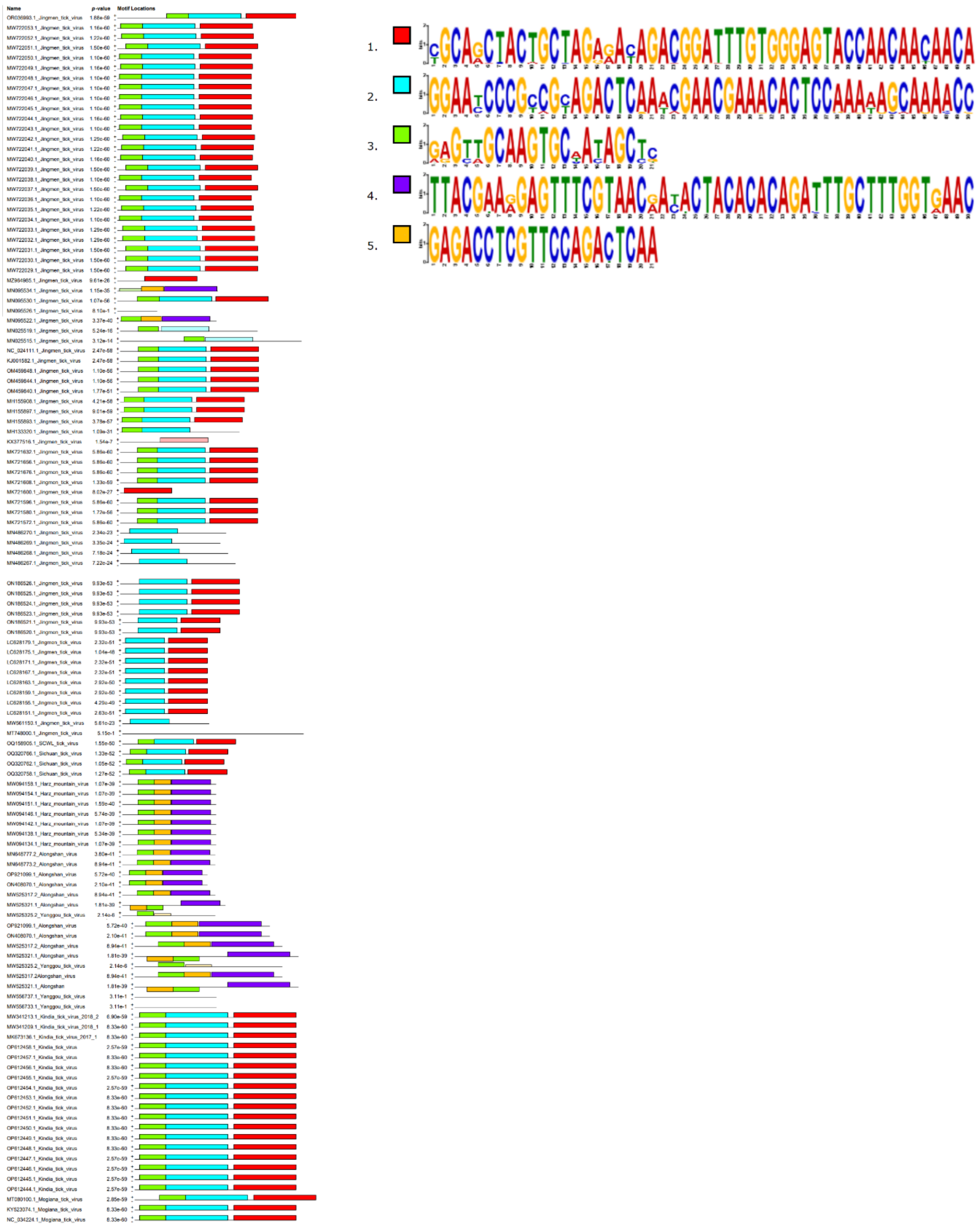

Figure S11. Conservative motifs in the 5′ UTR of segment 4 of segmented flavi-like viruses.

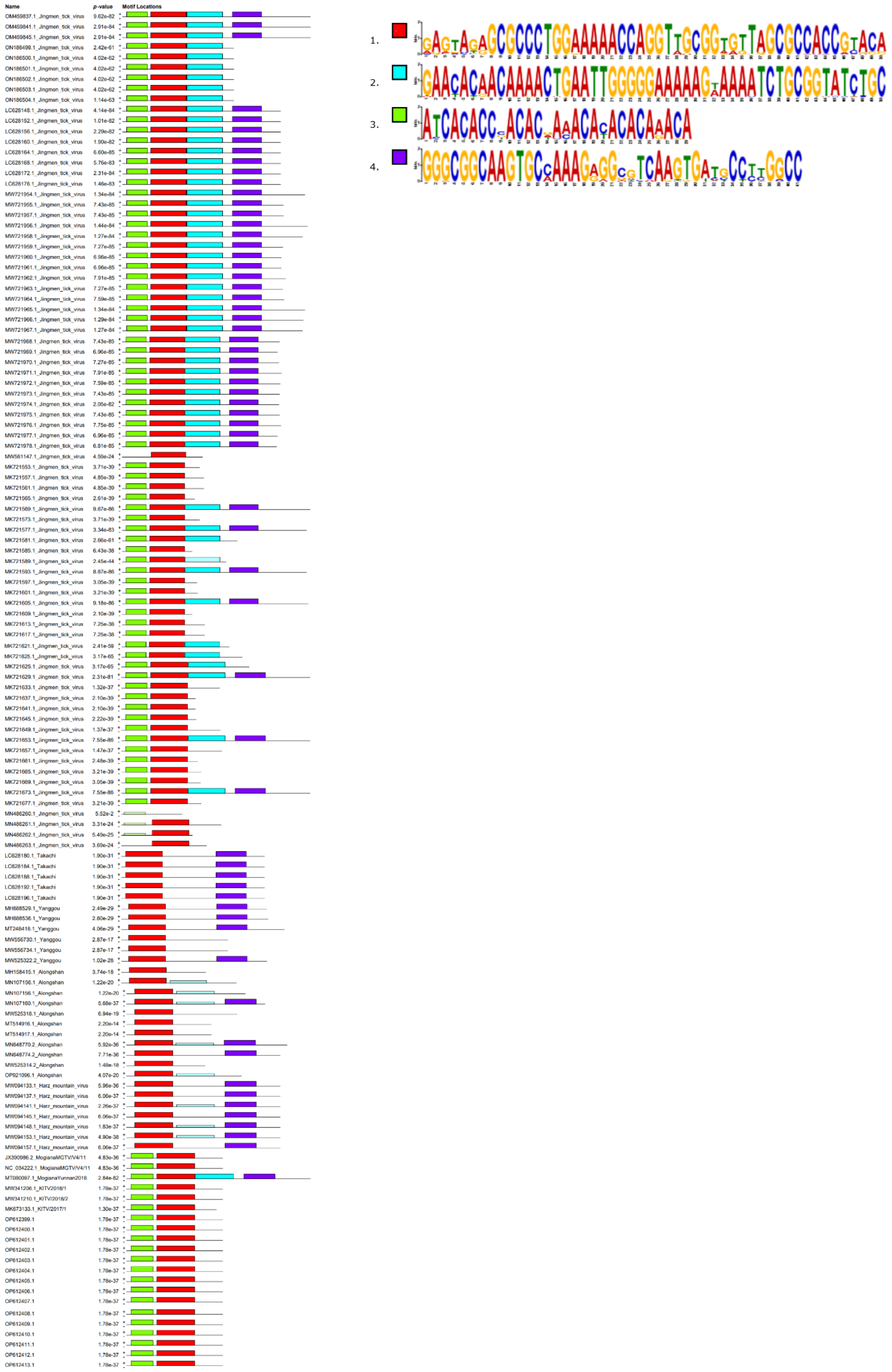

Figure S12. Conservative motifs in the 3′ UTR of segment 1 of segmented flavi-like viruses.

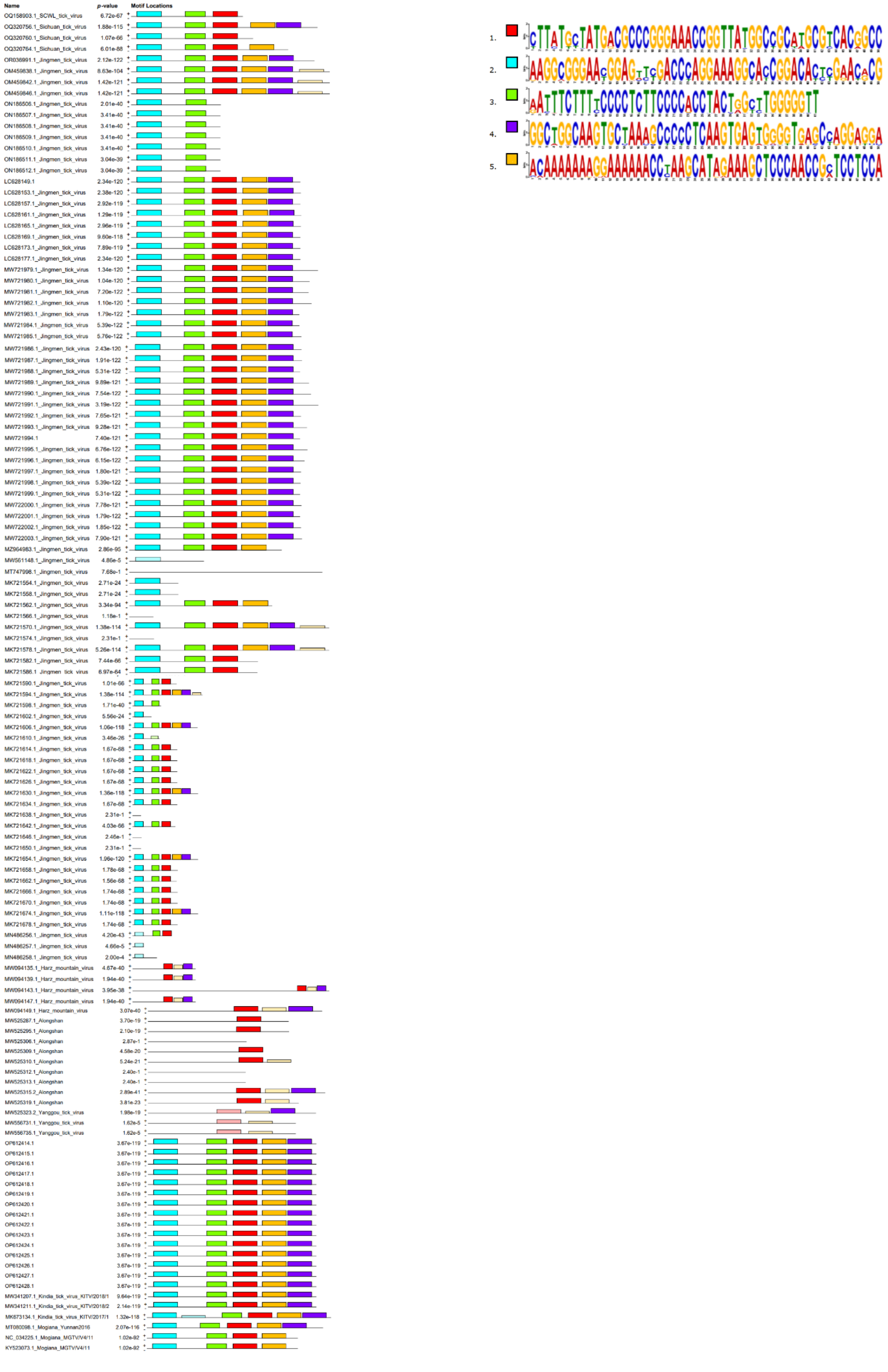

Figure S13. Conservative motifs in the 3′ UTR of segment 2 of segmented flavi-like viruses.

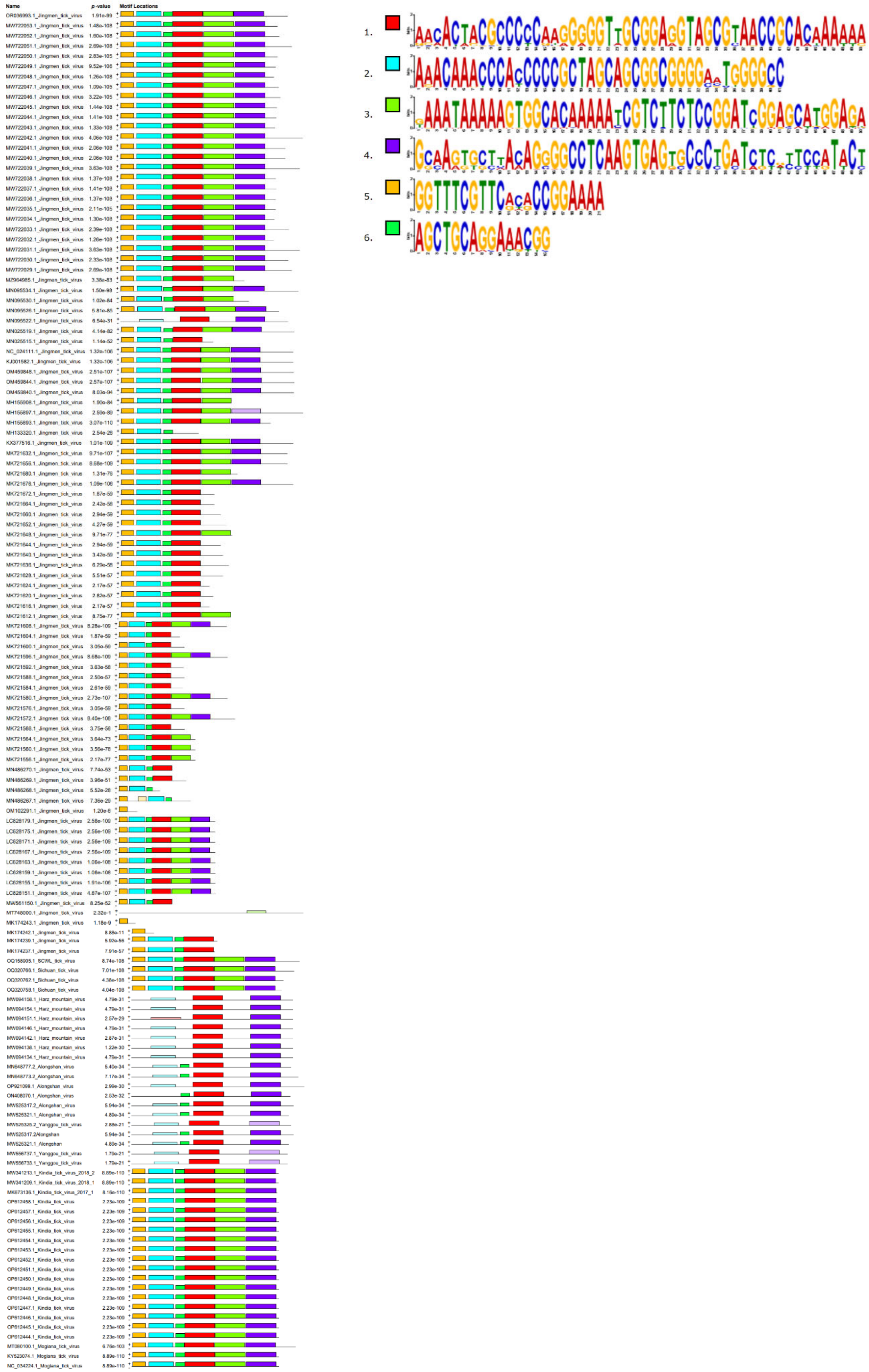

Figure S14. Conservative motifs in the 3′ UTR of segment 4 of segmented flavi-like viruses.
